# Flexible and Asymmetric Ligand in Constructing Coordinated Complexes: Synthesis, Crystal Structures and Fluorescent Characterization

**DOI:** 10.3390/ijms12010046

**Published:** 2010-12-27

**Authors:** Peng Chen, Jianhua Lin

**Affiliations:** Department of Osteology, The First Affiliated Hospital of Fujian Medical University, Fuzhou 350005, China; E-Mail: chenpengxy@yahoo.com.cn

**Keywords:** flexible and asymmetric ligand, double helical conformation, zigzag configuration

## Abstract

Flexible and asymmetric ligand L [L = 1-((pyridin-3-yl)methyl)-1*H*-benzotriazole], is used as a basic backbone to construct complicated metal-organic frameworks. Two new polymers, namely, [Ag_2_(L)_2_(NO_3_)_2_]*_n_* (**1**) and [Ag(L)(ClO_4_)]*_n_* (**2**), were synthesized and characterized by X-ray structure analysis and fluorescent spectroscopy. The complex **1** gives an “S” type double helical conformation, whereas complex **2** exhibits a 1D zigzag configuration. Different anions affect the silver coordination geometry and crystal packing topology.

## 1. Introduction

In the research of supramolecular chemistry, great interest has recently focused on crystal engineering of coordination frameworks due to their intriguing architectures, new topologies, intertwining phenomena and potential applications in microelectronics, nonlinear optics, ion exchange, molecular selection, molecular separation and recognition [1–8]. The structural motifs of coordination polymers rest on several factors, such as the central atom, the performance of the ligands, the coordinated and/or non-coordinated counter ions, the solvent systems and the reaction conditions. The choice of appropriate ligands is no doubt the key factor because it has an obvious influence on the topologies of the coordination polymers and behavior of the molecules. Some flexible bidentate ligands, which can adopt various conformations, have be widely used to construct helixes [9–12]. So far, a number of metal complexes utilizing flexible bis (triazole), bis (benzotriazole) or dipyridyl ligands have been reported [13–16], but the symmetry of these ligands has greatly limited the novelty and variety of the configuration.

**Figure 1:**
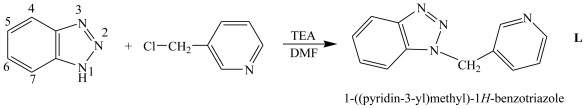
Synthesis and structure of L (1-((pyridin-3-yl)methyl)-1*H*-benzotriazole).

Recently, by a radical nucleophilic substitution, we obtained the hybrid heterocyclic ligand L (L = 1-((pyridin-3-yl)methyl)-1*H*-benzotriazole), which is a versatile N-donor in transition metal chemistry (Figure 1). From a structural point of view, it should be pointed out that (a) as a kind of angular ditopic ligand, the two exo-N atoms of its two different rings can μ_2_-bridge to two different metal atoms; (b) though the CH_2_ spacer‘s torsion angle can be twisted only within a limited range due to the *sp*^3^ configuration of C atom, its pyridine and benzotriazole rings bear a variable dihedral angle to meet the requirement of coordination geometries of metal ions as well as minimize steric interactions in the assembly process; (c) due to its unsymmetrical nature, L satisfies the fundamental requirements for the construction of acentric solids and NLO (non-linear optical) materials. Furthermore, we speculate that the non-coordinated counter anion‘s ligands may affect the polymers‘ structure and properties. In this paper, we have studied the interaction of L with silver nitrate and perchlorate. From our attempts, two new polymers, namely [Ag_2_(L)_2_(NO_3_)_2_]*_n_* (**1**) and [Ag (L)(ClO_4_)]*_n_* (**2**), were obtained as crystals suitable for single-crystal X-ray analysis.

## 2. Results and Discussion

### 2.1. Structure Description

**Figure 2:**
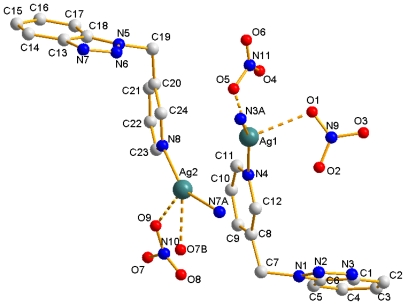
Drawing of two Ag(I) cations‘ coordination environment of the asymmetry unit in **1** [symmetry code. A: −x + 1/2, y + 1/2, −z + 1/2; B: −x + 1, −y + 1, −z].

The asymmetry unit of **1** consists of two independent Ag(I) cations, two L ligands, one integrated nitrate and two counteranion nitrate halves, as shown in Figure 2. The environment of every Ag center is the same tetrahedron geometry. Each Ag(1) center is coordinated by two L ligands via the N*_py_* [Ag(1)-N(4) = 2.238(4) Å] and N*_bta_* [Ag(1)-N(3A) = 2.217(4) Å] nitrogen donor atoms. For the balance of electric charge, each Ag(1) cation also displays very weak contact to two different nitrates (counteranion half) with O(1) and O(5) [Ag(1)-O(1) = 2.588(6) and Ag(1)-O(5) = 2.404(15) Å, respectively]. Each Ag(2) center adopts almost the same coordination mode as Ag(1), coordinated by two N from two L ligands and two O atoms form two different nitrates (counteranion integrated), with the Ag-N distances ranging from 2.221(4) to 2.241(4) Å, and Ag-O distances ranging from 2.404(15) to 2.588(6) Å.

**Figure 3:**
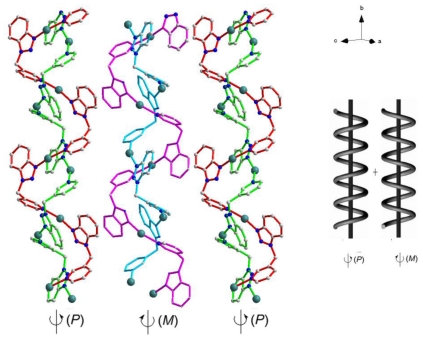
View of the “S” type double helical chains of **1**. The balanced packing of left (*P*) and right (*M*) chirality affords this packing and internal racemate.

In each L molecule, the *sp*^3^ configuration of C of -CH_2_- spacer forces the L ligand to be non-linear, with the N(1)-C(7)-C(8) angle 110.2(4)° and the N(5)-C(19)-C(20) angle 111.3(5)°. Each L behaves as an exo-bidentate linker, bridging adjacent Ag(I) cations to give rise to an [-Ag(L)-]*_n_* infinite 2^1^ helix strand, and such that each strand is interwind compactly by another [-Ag(L)-]*_n_* strand through the aurophilic d^10^-d^10^ interactions (Ag···Ag distance 3.2683 Å) to form a double helical species. Unlike the standard tubular double helixes that have been reported [17–19], the double helical chain of **1** wriggles with an “S” configuration, as shown in Figure 3. This result can be attributed to the 3- position of the pyridyl ring as the metal coordination site, which limits the stretched-out direction. Each chiral double helical chain links to its adjacent symmetric-related equivalents via the weak attraction of nitrate anion, to generate a quasi 3D supramolecular architecture. In each monocrystal, the balanced packing of left (*P*) and right (*M*) chains counteracts the chirality.

**Figure 4:**
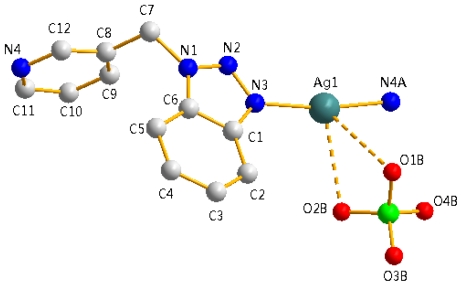
A drawing of an asymmetric unit of **2**. Symmetry codes: (**A**) x + 1, y, z−1; (**B**) x, y, z−1.

A drawing of the asymmetric unit of **2** is shown in Figure 4, with selected bond distances and angles listed in Table 1. The crystal structure reveals that the Ag(I) center is coordinated with two N atoms from two different L, with the Ag-N distances 2.135(3) and 2.113(3) Å, and the N(3)-Ag(1)-N(4) angle 169.97(10)°. The perchlorate group chelates to the Ag(I) center through very weak attraction, with Ag-O distances 2.905(7) and 3.022(6) Å, and the O(2B)-Ag(1)-O(1B) angle 44.99(14)°. For each L ligand in **2**, the N(1)-C(7)-C(8) angle is 111.3(2)° around the -CH_2_- spacer.

**Figure 5:**
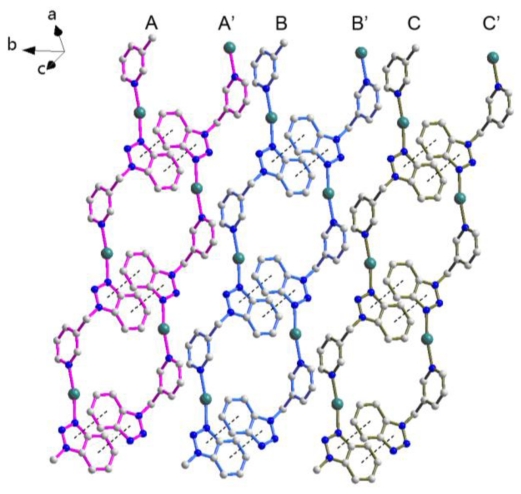
The infinite “Z” configuration of **2** and the dual π-π interactions between the dimers. Symmetry code: A: x + 1, y + 2, z + 2; A‘: −x + 3, −y + 3, −z + 1; B: x + 1, y + 1, z + 2; B‘: −x + 3, −y + 2, −z + 1; C: x, y, z + 3; C‘: −x + 2, −y + 1, −z + 2.

In **2**, each L group also behaves as an exo-bidentate linker, bridging adjacent Ag(I) cations to form an infinite “Z” configuration alone the [100] direction (Figure 5). Furthermore, each zigzag chain links to its adjacent symmetric-related equivalents via the duple π-π interactions with centroid to centroid distances 3.586(1) Å to generate a dimer. Other parameters of the π-π interactions are listed in Table 1. It is necessary to point out that these dual π-π interactions were not frequently observed in previously reported architectures [20,21].

### 2.2. Fluorescent Properties

**Figure 6:**
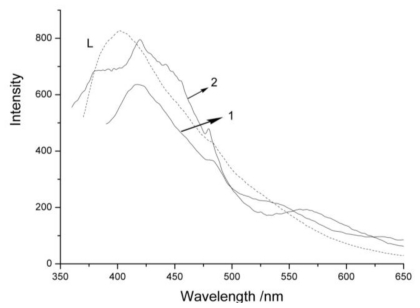
Fluorescent spectra of L (dashed line) and complex **1** and **2** in the solid state at room temperature.

Metal-organic polymer compounds with a d^10^ configuration have been found to exhibit photoluminescent properties [22–24]. Here, we wanted to examine the photoluminescence of **1** and **2**. The solid-state emission spectra of L and complexes **1** and **2** at room temperature are shown in Figure 6. It can be observed that the intense emissions occurr in the same range for the two complexes (λ_ex_ = 350 nm, λ_em_ = 417.5 nm for **1**; λ_ex_ = 340 nm, λ_em_ = 419.5 nm for **2**), which show a very light red-shift to that observed from L (λ_ex_ = 340 nm, λ_em_ = 402 nm). In d^10^-metal ions with one or two positive charges, the d-orbitals are contracted and therefore the electrons in these orbitals are much less accessible for back bonding to p-acceptor ligands. Moreover, silver cations have weak electro-accepting nature with respect to electrons from L, so these emissions can be assigned to ligand-to-metal charge transfer (LMCT) bands [25].

## 3. Experimental Section

### 3.1. Materials and Methods

Benzotriazole was purchased from Acros Ltd. Company and used without further purification, the other reagents were commercially available and used as purchased. The IR spectra as KBr discs were recorded on a Magna 750 FT-IR spectrophotometer. C, H, and N analysis were determined on an Elementary Vario ELIII elemental analyzer. Fluorescent spectra were measured on an Edinburgh Instruments analyzer model FL920.

### 3.2. Synthesis of the Ligand

The desired L was prepared by condensation of 1*H*-benzotriazole with the 3-picolyl chloride in DMF at reflux 4 h in the presence of triethylamine as basic catalyst [26]. Separation of pure L was performed by chromatography on a silica gel column (eluent ethyl acetate-light petroleum 40:60).

### 3.3. Synthesis of [Ag_2_(L)_2_(NO_3_)_2_]*n* (**1**)

A solution of L (0.021 g, 0.10 mmol) in MeOH (5 mL) was carefully layered on a solution of AgNO_3_ (0.017 g, 0.10 mmol) in H_2_O (5 mL). Diffusion between the two phases over a period of two weeks produced colorless block crystals. Yield: 0.012 g (32% based on L); Elementary analysis: calcd. for Ag_2_C_24_H_20_N_10_O_6_(760.24): C, 37.92; H, 2.65; N, 18.42%; found: C, 38.20; H, 2.89; N, 18.43%. IR (KBr, cm^−1^): 3700–3600 (w), 1605 (w), 1482 (w), 1496 (w), 1384 (s), 1227(s), 1194 (m), 1164 (m), 1099 (m), 950 (m), 821(m), 779 (m), 744 (s), 708 (m), 645 (m).

### 3.4. Synthesis of [Ag(L)(ClO_4_)]*n* (**2**)

The procedure of **2** is similar to the synthesis of **1** except that AgClO_4_ was used instead of AgNO_3_. Yield: 0.015 g (48% based on L); Elementary analysis: calcd. for AgClC_12_H_10_N_4_O_4_ (417.55): C, 34.52; H, 2.41; N, 13.42%; found: C, 34.37; H, 2.59; N, 13.65%. IR (KBr, cm^−1^): 3568(m), 1614(m), 1498 (w), 1459 (w), 1384 (s), 1312 (m), 1224 (m), 1166 (m), 1120 (w), 781 (w), 764 (w), 743 (m), 725(w), 626 (w), 563 (w), 483 (w), 433(w).

### 3.5. X-ray Crystallography

The single crystal X-ray diffraction measurements were carried out on a Siemens Smart CCD area detector. Intensities of reflections were measured using graphite monochromatized Mo-Kα radiation (*λ* = 0.71073 Å) with ω scan mode at 293(2) K in the range of 2.11° < *θ* < 27.48° for **1** and 2.42° < *θ* < 27.48° for **2**. Unit cell dimensions were obtained with least-squares refinements, and semi-empirical absorption corrections were applied using SADABS program [27]. The structure was solved by direct method [28] and non-hydrogen atoms were obtained in successive difference Fourier syntheses. The final refinements were performed by full-matrix least-squares methods on F^2^ by SHELXL-97 program package [29]. For both **1** and **2**, hydrogen atoms were generated geometrically and treated as riding. The crystallographic data for **1**–**2** are summarized in Table 2, and the selected bond distances and angles are listed in Table 3.

## 4. Conclusion

For L, the advantages of flexibility and exo-bidentate are fully demonstrated by the structural data. Two Ag(I) coordination polymers both possess 1d catenulate structure. It is even more surprising that different anions would lead to the complete diversity of the ultimate coordinated compounds‘ geometrical structures. However, to our disappointment, we failed to achieve some acentric solids or NLO materials as predicted. Some deeper reconstruction at the -CH_2_- spacer of the ligand is now being processed for achieving the chirality, and more tests with other metal cations (such as Pt^2+^ and Pd^2+^), counter anionic donors (such as *p*-toluenesulfonate and hexafluoro-phosphate), solvent systems and reaction conditions are in progress. It can be expected that many other novel asymmetry metal-organic materials will be realized.

## Supplementary Material

Crystallographic data for the structures reported in this paper have been deposited with the Cambridge Crystallographic Data Centre as supplementary publication No. CCDC 794748 for 1 and No. CCDC 794749 for 2. Copies of the data can be obtained free of charge on application to CCDC, 12 Union Road, Cambridge CB2 1EZ, UK (Fax: +44-1223-336-033; E-Mail: deposit@ccdc.cam.ac.uk).

## Figures and Tables

**Table 1:** Parameters of the π-π interactions in **2**.

	D_cc_ (Å)	D_pp_ (Å)	Dihedral angel (°)
Benzene to Triazole	3.586(1)	3.309(2)	1.38(19)
Triazole to Benzene	3.586(1)	3.275(1)	1.38(19)

**The Value of Ring Shifts (Å)**			

Benzene	2.400	triazole	2.694

**Table 2:** Crystallographic data for complexes **1** and **2**.

Compound	1	2
Formula	C_12_H_10_N_5_O_3_Ag	C_12_H_10_N_4_O_4_AgCl
Formula Weight	380.12	417.55
Crystal size (mm)	0.50 × 0.20 × 0.10	0.20 × 0.15 × 0.10
Crystal system	Monoclinic	Triclinic
Space group	P2_1_/*n*	P-1
*a* (Å)	12.0272(9)	9.1393(3)
*b* (Å)	14.0702(9)	9.4459(6)
*c* (Å)	15.8258(11)	10.0234(14)
*α* (º)	90	63.21(3)
*β* (º)	91.148(5)	65.45(3)
*γ* (º)	90	74.16(3)
*V* (Å^3^)	2677.6(3)	698.64(25)
*Z*	4	2
*D_c_* (Mg·m^−3^)	1.886	1.985
μ (mm^−1^)	1.524	1.658
*F* (000)	1504	412
*T* (K)	293(2)	293(2)
Reflns. Collected	6095	3163
Reflns. Unique	5012	2415
Parameters	409	199
Goodness-of-fit on *F*^2^	1.002	1.000
*R*_1_, _w_*R*_2_ [*I* > 2σ (*I*)]^a^	0.0532, 0.1188	0.0358, 0.0819
*R*_1_, _w_*R*_2_ (all data)^b^	0.0667, 0.1255	0.0524, 0.0908
Max, Min Δρ (e·A^−3^)	0.691, −0.714	0.572, −0.527

a R = ∑||F_0_| − |F_c_||)/∑|F_0_|;

b _w_R = [∑w (F_0_^2^ − F_c_^2^)^2^/∑w (F_0_^2^)^2^]^1/2^.

**Table 3:** Selected bond lengths (Å) and angles (°) for complexes **1** and **2**.

**1**			
Ag(1)-N(3A)	2.217(4)	Ag(2)-N(8)	2.241(4)
Ag(1)-N(4)	2.238(4)	Ag(2)-N(7A)	2.221(4)
Ag(1)-O(1)	2.588(6)	Ag(2)-O(7B)	2.532(18)
Ag(1)-O(5)	2.404(15)	Ag(2)-O(9)	2.471(16)
N(3A)-Ag(1)-N(4)	156.26(18)	N(7A)-Ag(2)-N(8)	152.66(17)
N(1)-C(7)-C(8)	110.2(4)	N(5)-C(19)-C(20)	111.3(5)
Symmetry codes: (A) −x + 1/2, y + 1/2, −z + 1/2; (B) −x + 1, −y + 1, −z.			

**2**			
Ag(1)-N(3)	2.113(3)	Ag(1)-N(4A)	2.135(3)
Ag(1)-O(1B)	3.022(6)	Ag(1)-O(2B)	2.905(6)
N(3)-Ag(1)-N(4A)	169.97(10)	N(1)-C(7)-C(8)	111.3(2)
Symmetry codes: (A) x + 1, y, z−1; (B) x, y, z−1.			
